# Efficacy of thunder-fire moxibustion for cancer-related fatigue in breast cancer survivors: study protocol for a randomized controlled trial

**DOI:** 10.3389/fonc.2025.1496741

**Published:** 2025-03-11

**Authors:** Huakang Li, Zhonglin Zhang, Qiang Li, Yuyang Jin, Yunjing Jia, Pengxuan Gu, Qi Xiao, Lingna Jin, Ziliang Wu, Bing Lin, Shanshan Wei, Jinyi Lang

**Affiliations:** ^1^ School of Clinical Medicine, Chengdu University of Traditional Chinese Medicine, Chengdu, Sichuan, China; ^2^ Department of Oncology, Hospital of Chengdu University of Traditional Chinese Medicine, Chengdu, Sichuan, China; ^3^ Department of Radiation Oncology, Radiation Oncology Key Laboratory of Sichuan Province, Sichuan Clinical Research Center for Cancer, Sichuan Cancer Hospital & Institute, Sichuan Cancer Center, Affiliated Cancer Hospital of University of Electronic Science and Technology of China, Chengdu, Sichuan, China; ^4^ Institute of Sports Medicine and Health, Chengdu Sport University, Chengdu, Sichuan, China; ^5^ Health Management Center, Hospital of Chengdu University of Traditional Chinese Medicine, Chengdu, Sichuan, China

**Keywords:** moxibustion, cancer-related fatigue, breast cancer, randomized controlled trial, clinical trial protocol

## Abstract

**Background:**

Cancer-related fatigue (CRF) is one of the most prevalent and debilitating symptoms experienced by breast cancer survivors, often associated with dysregulation of the hypothalamic-pituitary-adrenal (HPA) axis. In China, moxibustion is widely used as a therapeutic approach for managing fatigue. Thunder-fire moxibustion (TFM), a novel technique with high thermal radiation and strong penetrative properties, may provide benefits for CRF. This study aims to assess the efficacy, safety, and underlying mechanisms of TFM in the treatment of CRF among breast cancer survivors.

**Methods:**

This prospective, single-center, open-label, randomized controlled trial will recruit 70 breast cancer survivors diagnosed with CRF. Participants will be randomly assigned in a 1:1 ratio to either a waitlist control group or a TFM intervention group. All participants will receive standard care during the 30-day treatment period. Those in the TFM group will additionally undergo TFM treatment every other day, totaling 15 sessions. The primary outcome measure is the change in total fatigue score, assessed using the Piper Fatigue Scale, from baseline to the end of treatment. Additionally, this study will investigate the underlying mechanisms of TFM by evaluating changes in HPA axis-related hormone levels, inflammatory markers, gut microbiota composition, and conducting metabolomic analyses of fecal and blood samples.

**Discussion:**

This study takes a multidisciplinary approach to comprehensively explore how TFM modulates biological systems involved in CRF, aiming to generate robust evidence. If successful, this study will provide high-quality, evidence-based reference points for the treatment of CRF in breast cancer survivors and inform future research in integrative medicine.

**Trial registration:**

The study has been registered with the International Traditional Medicine Clinical Trial Registry (http://itmctr.ccebtcm.org.cn, ITMCTR2024000406).

## 1 Introduction

The National Comprehensive Cancer Network defined cancer-related fatigue (CRF) as “a distressing, persistent, subjective sense of physical, emotional, and/or cognitive tiredness or exhaustion related to cancer or cancer treatment that is not proportional to recent activity and interferes with functioning” (1). Recognized as one of the most common and burdensome symptoms, CRF affects over half of all cancer patients (2). According to the latest data from GLOBOCAN 2020, breast cancer, with an estimated 2.3 million new cases, has overtaken lung cancer as the most frequently diagnosed cancer worldwide (3). Notably, CRF is more prevalent among patients with breast cancer, showing a higher incidence rate than in other cancer types (4). Over 90% of breast cancer patients experience CRF during or after treatment (5, 6), and approximately one-third of these patients suffer from moderate to severe levels of CRF (7). Current treatment options for CRF include primarily pharmacological and non-pharmacological schemes (8). Non-pharmacological treatments, including exercise, psychological support, cognitive-behavioral therapy, and sleep management, are the mainstay, yet their efficacy is often limited. Pharmacological treatments typically involve stimulants and corticosteroids, yet their use is often limited due to associated side effects. Consequently, the imperative to explore and implement new strategies to alleviate CRF in breast cancer patients, with the goal of improving their quality of life, has become a critical area of concern.

Moxibustion, a revered external treatment modality in traditional Chinese medicine, boasts a history exceeding 2,500 years (9). This technique, which entails burning moxa to stimulate specific acupuncture points, is widely employed across Asia for symptom management in cancer patients (10). According to the principles outlined in the classic Chinese medical text “The Inner Canon of the Emperor”, the theory of “warming the deficient” suggests that conditions characterized by deficiencies in yin and yang, such as CRF, should be treated with warmth. Moxibustion, based on the theory of meridians, aims to enhance the circulation within the meridian system, thereby warming and supplementing the vital energy (qi) and balancing yin and yang (11). Throughout ancient Chinese medical literature, it has been extensively documented as an effective method for treating fatigue. Recent meta-analyses further support its benefits in alleviating CRF symptoms (12, 13). Among the various moxibustion techniques, the most widely applied in current CRF research is traditional mild moxibustion. Other notable methods include governor vessel moxibustion and laser moxibustion. Governor vessel moxibustion offers the advantage of stronger thermal radiation, greater penetration, and broader coverage compared to mild moxibustion (14). However, it is more complex to perform, requiring multiple auxiliary tools and is associated with higher costs. In contrast, laser moxibustion mimics the heat radiation effect of moxibustion, offering precise, stable, and adjustable thermal radiation (15). However, it is important to note that traditional moxibustion’s combustion effects may involve multiple pathways, such as aromatic and herbal components (16).

Thunder-fire moxibustion (TFM), an innovative moxibustion therapy, was developed by Professor Zhao Shibi, drawing from her extensive medical experience spanning several decades (17). Currently, TFM is widely applied for musculoskeletal, respiratory, and neurological disorders (18). Preliminary meta-analytic evidence supports its efficacy in conditions such as back pain (19), knee osteoarthritis (20), allergic rhinitis (21), and postherpetic neuralgia (22). TFM, similar to governor vessel moxibustion, demonstrates high thermal radiation, deep penetration, and broad coverage (23). However, it offers superior cost-effectiveness, a simplified procedure, and eliminates the need for auxiliary tools. Compared to laser moxibustion, TFM does not require specialized equipment, making it more accessible and potentially effective through multiple mechanisms. Given the existing body of moxibustion research and the unique advantages of TFM, we hypothesize that TFM may prove to be an effective, economical, and accessible treatment for CRF.

To preliminarily explore the efficacy of TFM, we conducted a pilot study on breast cancer survivors who had completed cancer treatment and were in long-term follow-up. The selection of this specific cohort was based on practical feasibility. If CRF patients undergoing cancer treatment were chosen, perfect coordination with oncologists would be necessary, introducing complexity and additional confounding factors to the trial. Furthermore, considering the potential for cancer treatments to cause adverse events (AEs) and TFM’s potential to worsen these effects, this therapy was cautiously introduced only after fully establishing the safety of TFM. In this pilot study, 30 breast cancer survivors suffering from CRF randomly assigned to a moxibustion group (standard care + TFM every other day) and a control group (standard care). At the end of the 30-day treatment period, the total fatigue scores on the Piper Fatigue Scale (PFS) for the moxibustion group and the control group decreased by 1.93 points and 0.94 points, respectively, compared to baseline. No dropouts or treatment-related adverse events occurred in either group. Based on the feasibility and safety demonstrated by the pilot study results, the current study is designed to conduct a comprehensive randomized controlled trial to delve deeper into the therapeutic efficacy and safety profile of TFM for this patient demographic. Additionally, this study will explore the mechanism of action of TFM by analyzing changes in hypothalamic-pituitary-adrenal (HPA) axis-related hormone factors, inflammatory markers, gut microbiota, and conducting metabolomic analyses of fecal/blood samples.

## 2 Methods

### 2.1 Study design

**Figure 1:**
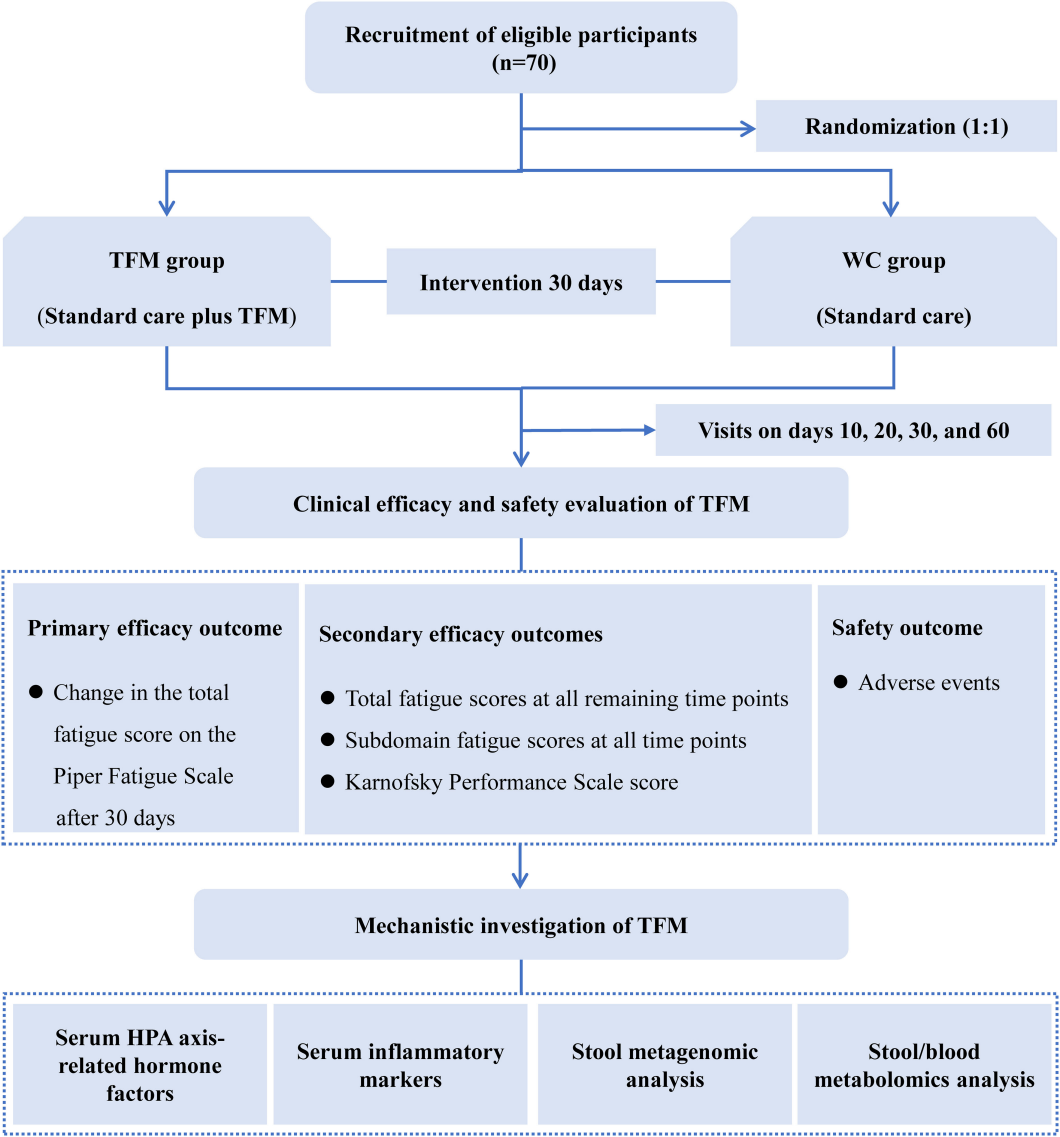
Study flow diagram. HPA, hypothalamic-pituitary-adrenal; TFM, thunder-fire moxibustion; WC, waitlist control. Serum HPA axis-related hormones include adrenocorticotropic hormone, corticotropin-releasing hormone, and cortisol. Serum inflammatory markers include interleukin-6 and interleukin-8.

The study is designed as a prospective, single-center, open-label, parallel-group, randomized controlled clinical trial. It aims to evaluate the efficacy and safety of TFM treatment in breast cancer survivors with CRF and to preliminarily explore its potential mechanisms. The study duration is 65 days, including a 5-day screening period, a 30-day treatment period, and a 30-day follow-up period. We plan to enroll 70 participants at the Hospital of Chengdu University of Traditional Chinese Medicine, who will be randomly assigned in a 1:1 ratio to either the waitlist control (WC) group or the TFM group. During the 30-day treatment period, all patients will receive standard care. Patients in the TFM group will receive additional TFM treatment every other day, for a total of 15 sessions. The primary outcome measure is the change in the total fatigue score on the PFS at the end of treatment compared to baseline. The study flow is illustrated in **Figure 1**.

### 2.2 Participants

#### 2.2.1 Recruitment

Participants will be recruited through the posting of flyers on the bulletin boards at the Hospital of Chengdu University of Traditional Chinese Medicine, as well as through social media outreach (hospital website, WeChat, Weibo). Eligibility for the study will be jointly determined by a licensed oncologist and an acupuncturist, based on inclusion and exclusion criteria. Researchers will inform potential participants about the purpose of the study, its requirements, the interventions involved, and all potential risks, ensuring that they fully understand the study parameters before providing their signed informed consent.

#### 2.2.2 Inclusion criteria

(1) Meets the International Classification of Diseases, 10th Revision (ICD-10), diagnostic criteria for CRF;
(2) Moderate to severe fatigue, defined as a score of ≥4 on the PFS;
(3) Diagnosed with stage I-III breast cancer in women;
(4) Age between 18 and 80 years old;
(5) Karnofsky Performance Status (KPS) score of ≥70;
(6) Completion of breast cancer treatment (surgery, radiotherapy, chemotherapy, targeted therapy) at least 12 weeks prior, but ongoing endocrine therapy exceeding 4 weeks is allowed.

#### 2.2.3 Exclusion criteria

(1) Pregnancy or breastfeeding;
(2) Anemia (hemoglobin < 110g/L);
(3) Abnormal thyroid function tests (free thyroxine > 22pmol/L, thyroid-stimulating hormone > 5.0uIU/ml);
(4) Abnormal liver function tests (elevated aspartate aminotransferase or alanine aminotransferase levels greater than twice the upper limit of normal, or bilirubin levels exceeding the upper limit of normal, including total, direct, or indirect bilirubin);
(5) Abnormal renal function tests (creatinine more than 1.5 times the upper limit of normal);
(6) Patients with psychiatric disorders (including but not limited to depression, schizophrenia, bipolar disorder) or severe cognitive impairment (e.g., significant difficulties with memory, attention, or executive functions) that affect the ability to fully understand the study content or comply with the study protocol.

#### 2.2.4 Drop-out criteria

(1) Participants have the right to voluntarily withdraw from the trial at any stage for any reason;
(2) Participants who do not formally declare their withdrawal but independently cease the prescribed treatment or participation in related examinations, or cannot be contacted during the follow-up period, will be considered to have substantively withdrawn from the trial;
(3) Participants who experience severe AEs that preclude continuation in the trial;
(4) Participants who show poor compliance, with fewer than 12 TFM sessions;
(5) Participants who use CRF treatments outside the study protocol (e.g., stimulants or corticosteroids).

### 2.3 Interventions

All patients will receive the standard care for CRF as recommended by the Oncology Branch of the Chinese Medical Association (24). The specific regimen will include: 1) Psychological support: imparting knowledge on breast cancer recovery and CRF, and engaging in weekly therapeutic communication with the patients, each session lasting approximately 2 hours, to assist in managing adverse emotions; 2) Nutritional management: conducting a nutritional risk assessment once a week using the Nutritional Risk Screening 2002 (NRS 2002) tool, with patients identified at nutritional risk being referred to the nutritional department for specialized treatment; 3) Sleep management: overseeing the establishment and maintenance of regular sleeping schedules and environments conducive to sleep, with pharmacological treatment being administered as necessary; 4) Exercise guidance: supervising patients to partake in 180 to 300 minutes of moderate-intensity physical activities per week, such as brisk walking, yoga, and Tai Chi. During the study, the use of treatments outside the study protocol for CRF will be prohibited (e.g., stimulants or corticosteroids).

The TFM group will receive TFM therapy. The moxa sticks used for the treatment are produced by Chongqing Zhao’s Thunder-Fire Moxibustion Traditional Medicine Research Institute in China, each measuring 2.8 cm in diameter, 10.5 cm in length, and weighing 230 g. TFM will be carried out by four female acupuncturists, each with more than three years of experience in moxibustion therapy and holding a professional license. The acupoints for treatment will include CV6 (Qihai), CV4 (Guanyuan), SP6 (Sanyinjiao), and both sides ST36 (Zusanli). The acupuncturists will manipulate a lit moxa stick, moving it up and down at a vertical distance of 2-3cm from the skin over the acupoints for 30 minutes, inducing a sensation of deep tissue warmth without causing any burn pain. The treatment frequency will be every other day for 30 days, totaling 15 sessions. The WC group is scheduled to commence TFM therapy following the conclusion of the study.

### 2.4 Outcomes

#### 2.4.1 Primary efficacy outcome

The primary efficacy outcome is the change in the total fatigue score on the PFS at the end of a 30-day treatment period relative to baseline. The PFS is a well-validated, multidimensional tool used to assess the severity of CRF and is highly sensitive to fluctuations in fatigue levels over time (14). It has been widely utilized in clinical trials assessing CRF interventions in breast cancer survivors (25, 26) thereby enhancing the external validity and comparability of our study results. The PFS consists of 22 rating items, which are divided into four subdomain scales: behavioral fatigue (6 items), emotional fatigue (5 items), sensory fatigue (5 items), and cognitive fatigue (6 items), with each item scored on a scale from 0 to 10. The total fatigue score is the average of these 22 items, with severity categorized as mild (1-3 points), moderate (4-6 points), and severe (7-10 points). Assessments are conducted at baseline, on days 10, 20, and 30 (end of treatment), and on day 60 (end of follow-up).

#### 2.4.2 Secondary efficacy outcomes

The critical secondary efficacy endpoints include changes in total fatigue scores at all other measurement time points (day 10, day 20, and day 60) compared to baseline, as well as changes in the four subdomain fatigue scores at all time points (day 10, day 20, day 30, and day 60) relative to baseline. Another secondary endpoint is the quality of life, which will be assessed using the KPS. The KPS ranges from 0 to 100, with higher scores indicating a better quality of life.

#### 2.4.3 Mechanism outcomes

To investigate the underlying mechanisms of action of TFM, this study will collect blood and fecal samples from all participants at baseline and after a 30-day treatment period. Fasting blood samples will be collected between 7:00 AM and 9:00 AM, and the levels of serum HPA axis-related hormones, including adrenocorticotropic hormone (ACTH), corticotropin-releasing hormone (CRH), and cortisol (CORT), as well as serum inflammatory markers, including interleukin-6 (IL-6) and interleukin-8 (IL-8), will be assessed using enzyme-linked immunosorbent assay (ELISA). The gut microbiota in fecal samples will be analyzed through 16S rRNA gene sequencing. Furthermore, untargeted metabolomics analyses of serum and fecal samples will be performed using liquid chromatography-mass spectrometry (LC-MS) technology. A variety of bioinformatics approaches will also be employed for in-depth analysis of the data, including but not limited to alpha and beta diversity analysis, principal component analysis (PCA), principal coordinates analysis (PCoA), non-metric multidimensional scaling (NMDS), linear discriminant analysis effect size (LEfSe), and partial least squares discriminant analysis (PLS-DA).

### 2.5 Safety assessment

Throughout the study, meticulous documentation will be maintained for all AEs encountered. This documentation will include comprehensive details such as treatment group allocation, time of occurrence, duration, severity, management interventions, and outcomes. The severity of AEs will be assessed using the Common Terminology Criteria for Adverse Events (CTCAE) version 5.0 (27), published by the National Institutes of Health (NIH), with grades 3-5 classified as severe AEs. Potential AEs associated with TFM may include, but are not limited to, xerostomia, dizziness, respiratory symptoms, scalding sensations, itching, and fainting.

### 2.6 Participant timeline

**Figure 2:**
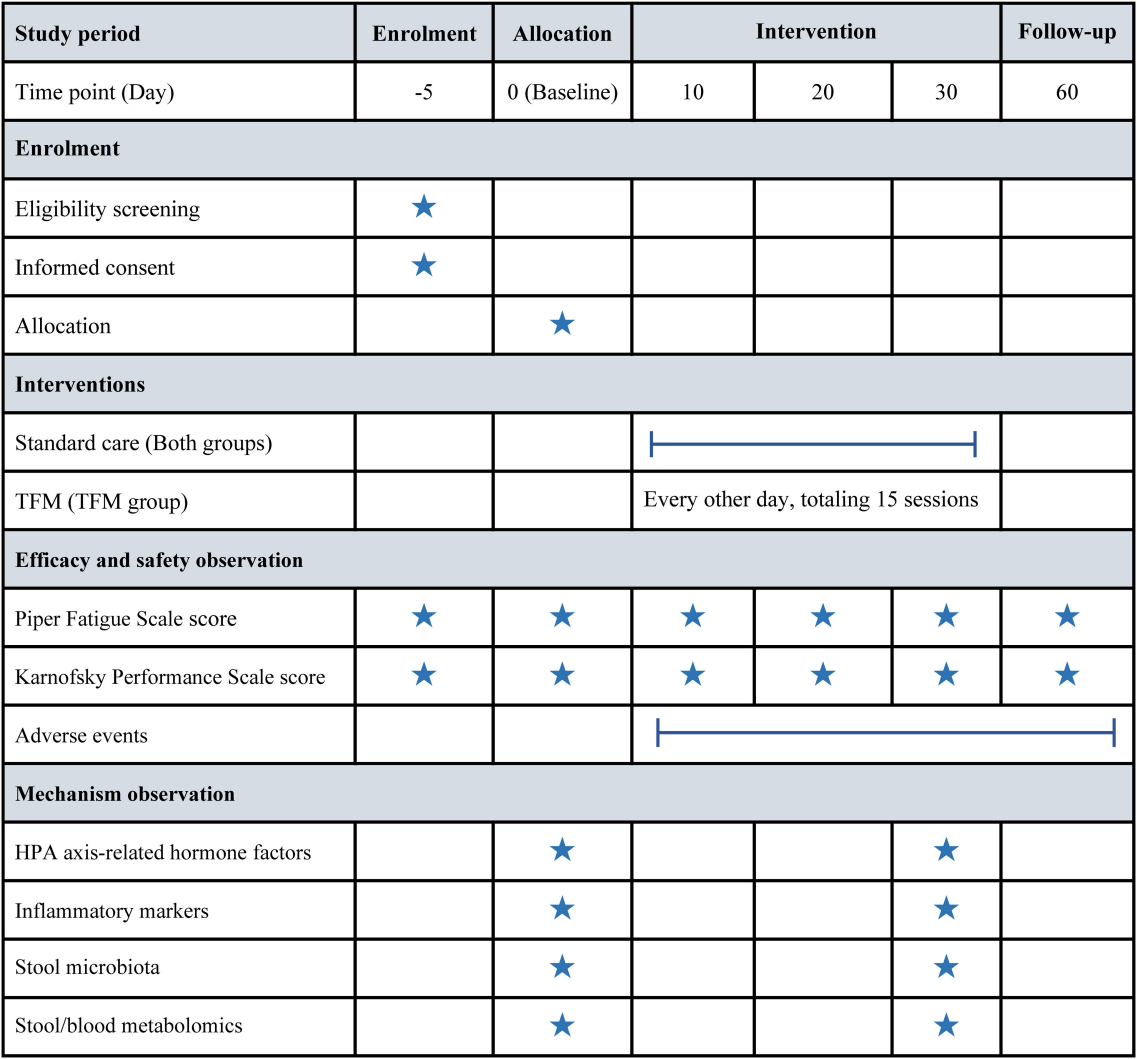
Timeline of enrollment, intervention, and assessment milestones. HPA, hypothalamic-pituitary-adrenal; TFM, thunder-fire moxibustion.

**Figure 2** presents a detailed timeline, illustrating the comprehensive schedule for the measurement of outcomes and the systematic collection of data.

### 2.7 Sample size

The sample size was calculated based on the primary efficacy outcome, using data from a preceding pilot study. At the end of the 30-day treatment period, total fatigue scores in the TFM and WC groups decreased by 1.93 ± 0.47 and 0.94 ± 0.35 points, respectively, compared to baseline. The sample size calculation was conducted using the “Two-Sample T-Tests for Superiority by a Margin Allowing Unequal Variance” module in PASS software (version 15.0). Assuming a 20% dropout rate, a one-sided α of 0.025, a statistical power (1-β) of 0.99, and a superiority margin of 0.5, it was determined that at least 35 patients should be allocated to each group. Since the primary efficacy outcome will be analyzed using a linear mixed model, which offers greater statistical power than the traditional two-sample t-test (28), the larger sample size derived from the t-test calculation will further enhance the robustness and reliability of the analysis.

### 2.8 Randomization and blinding

An independent statistician will use a variable block size randomization method with block sizes of 4, 6, and 8 to create a random sequence, ensuring a balanced 1:1 distribution between the TFM group and the WC group. The specifics of the group assignments will be noted on cards, which will then be sealed in opaque envelopes and stored by a third party. After completing the initial assessments, patients will sequentially open these envelopes in the order of their enrollment to discover their assigned group.

Given the novelty and uniqueness of TFM therapy, no placebo tool has yet been developed for TFM, making the implementation of a double-blind design challenging. Consequently, the study will be conducted as open-label, with the knowledge of group assignments transparent to both patients and acupuncturists. To minimize potential biases, those involved in evaluating outcomes, handling data, and performing statistical analysis will be kept unaware of the group allocations.

### 2.9 Statistical analysis

All analyses will be conducted within the intention-to-treat population, encompassing all randomized participants. For continuous variables, data adhering to a normal distribution will be expressed as mean ± standard deviation, while non-normally distributed data will be presented using the median and interquartile range. The Shapiro-Wilk test will be used to determine the normality of the data. Categorical variables will be depicted as frequencies and percentages. The planned statistical analyses will be conducted using SPSS software (version 28.0) and the R programming language (version 4.3.1). Significance testing will be bilateral, with a P-value of ≤ 0.05 denoting statistical significance.

To compare the longitudinal trajectories of changes in PFS scores from baseline between groups at successive follow-up time points, linear mixed models will be employed. Given the correlation between repeated measures within the same participant, this model will include participant-specific random effects (29). The fixed effects of the model will include group, follow-up time, and the interaction between group and follow-up time (30). Covariates will include age and baseline PFS scores. No imputation of missing data is planned, as the analysis model itself accounts for missing data and remains valid under the missing-at-random assumption.

For other continuous outcome measures, such as KPS scores, HPA axis-related hormonal factors, and inflammatory markers, comparison between groups will employ analysis of covariance adjusting for baseline values if the data are normally distributed. If not, the Mann-Whitney U test will be applied. Missing data will be addressed through the application of the last observation carried forward method.

We plan to conduct predefined subgroup analyses for the primary outcome, considering the following baseline variables: age (≤55, >55), nutritional risk (no, yes), sleep disorder (no, yes), fatigue severity (moderate, severe), body mass index (≤24, >24), KPS score (70, 80, 90, 100), cancer stage (I, II, III), cancer subtype (Luminal A, Luminal B, HER2-positive, triple-negative), and cancer treatment (surgery, radiotherapy, chemotherapy, targeted therapy, endocrine therapy). Consequently, except for the primary outcome, the interpretations of other outcomes and subgroup analyses will be treated as exploratory.

### 2.10 Quality control and data management

Prior to initiating recruitment for the study, it will be imperative that all researchers undergo specialized training programs. These programs will be tailored to ensure the seamless conduct of the study and to solidify understanding of trial management protocols. The training will encompass various key aspects of this trial, such as trial design, inclusion and exclusion criteria, intervention methods, outcome measurement, reporting of AEs, and the documentation in case report forms. TFM operations will be conducted by female acupuncturists who have more than three years of moxibustion experience and hold a traditional Chinese medicine practitioner’s license.

For data acquisition, paper-based case report forms will be utilized to meticulously document each participant’s demographic information and assessment outcomes. All paper-based records will be securely stored in locked filing cabinets by the study investigators. The electronic data will be entered by two experienced and independent data entry personnel using a double-entry method and will be stored in a secure, encrypted study folder accessible only to the study team. Our team will be committed to upholding the confidentiality of all data, ensuring protection against any potential breaches or losses. The research ethics committee of Chengdu University of Traditional Chinese Medicine will conduct regular audits to monitor the trial’s data, oversee its execution, evaluate its progress, and ensure its adherence to established standards and protocols.

### 2.11 Trial status

Recruitment for this clinical trial is expected to begin in October 2024.

## 3 Discussion

This randomized controlled clinical trial aims to evaluate the clinical efficacy and safety of TFM treatment in breast cancer survivors with CRF. In addition, the study will preliminarily explore the potential mechanisms of TFM by investigating whether it can modulate changes in inflammatory markers, gut microbiota, and the fecal/blood metabolic profile. These changes may influence HPA axis-related hormone factors, ultimately leading to the alleviation of CRF.

The HPA axis, a key component of the neuroendocrine system, regulates ACTH secretion through positive feedback mechanisms during normal stress responses, which in turn promotes CORT release. This process is crucial for maintaining immune system homeostasis and metabolic balance (31). Several studies have suggested that decreased CORT levels, combined with elevated ACTH secretion due to negative feedback mechanisms, form the physiological basis of CRF (32, 33). A retrospective clinical study found that a 4-week regimen of mild moxibustion treatment could alleviate chronic fatigue syndrome by enhancing CORT levels (34). Furthermore, inflammation is considered a critical factor in HPA axis dysfunction. Research shows that tumors and their treatments can activate pro-inflammatory cytokine networks (35). These inflammatory factors may reach the brain through various pathways, impairing CORT synthesis and release, thus contributing to fatigue (36). A study involving 108 breast cancer survivors found a positive correlation between CRF and elevated levels of IL-6 and IL-8 (37), highlighting the potential inflammatory underpinnings of this condition. A randomized controlled trial demonstrated that a 4-week course of TFM treatment could alleviate knee osteoarthritis symptoms by reducing IL-6 and tumor necrosis factor levels (38). These findings support the hypothesis that TFM may alleviate CRF through modulation of the HPA axis and inflammatory pathways.

The gut microbiota is recognized as a key mediator between the gastrointestinal and central nervous systems (39), with substantial evidence supporting its role in CRF. Research indicates that patients with milder CRF tend to have a higher relative abundance of short-chain fatty acid-producing bacteria with anti-inflammatory effects, while those with more severe CRF have an increased presence of inflammation-related bacteria (40). Moreover, an imbalance in gut microbiota can impair tight junction proteins, leading to increased gut permeability, systemic inflammation, and a weakened blood-brain barrier (41). This cascade can provoke central inflammatory responses that may worsen fatigue. Notably, a clinical trial demonstrated that a 4-week course of governor vessel moxibustion therapy may improve the symptoms of ankylosing spondylitis by increasing the abundance of *Lactobacillus*, a beneficial bacterium with anti-inflammatory properties (42). Metabolomics has been instrumental in identifying biomarkers that capture the body’s metabolic status and reactions to physiological shifts. Given that the gut microbiota serves as a major source of human metabolites, changes in its composition can profoundly affect metabolic signaling (43). Importantly, research shows that serum metabolite profiles differ significantly between CRF patients and those without fatigue, underscoring the influence of metabolic dysfunction in CRF (44). An animal study suggested that moxibustion exerts anti-fatigue effects, potentially through modulation of the gut microbiota and metabolic pathways, with increased levels of *Bacteroides* and decreased concentrations of lithocholic acid identified as key targets (45). These findings collectively support the hypothesis that TFM may alleviate CRF through its modulatory effects on both gut microbiota composition and metabolic pathways.

The use of multiple acupoints in combination may enhance therapeutic efficacy. In clinical practice, acupoint stimulation for CRF typically involves the simultaneous application of several acupoints to achieve a synergistic effect and more comprehensive therapeutic outcomes. In this study, CV6 (Qihai), CV4 (Guanyuan), SP6 (Sanyinjiao), and bilateral ST36 (Zusanli) were selected as the primary treatment acupoints. This selection is based on a data mining study on acupoint stimulation for CRF (46), which revealed that these acupoints form a core combination for effective CRF treatment. According to traditional Chinese medicine theory, the pathogenesis of CRF is often associated with Qi deficiency (insufficient vital energy). These four acupoints are known for their ability to tonify Qi and replenish deficiency, thereby helping to regulate Qi and blood and enhance the body’s restorative capacity (46). Nevertheless, further research is necessary to compare the effects of single versus multiple acupoint treatments for CRF. Such studies would provide valuable insights into optimizing acupoint selection, ultimately guiding clinical practice.

This study adopts a multidisciplinary approach to thoroughly investigate how TFM modulates biological systems associated with CRF, aiming to build a robust evidence base. However, several limitations must be acknowledged. First, as a single-center trial, the findings require validation in larger, more diverse populations. Second, challenges related to blinding and placebo effects, common in moxibustion studies, may introduce bias. Future studies should focus on developing a placebo tool specific to TFM and conducting double-blind trials to more accurately assess its efficacy. Lastly, the study does not examine certain CRF-related biomarkers, such as serotonin, which may be crucial for fully understanding the biological changes involved in CRF.

## 4 Ethics and dissemination

This study adheres strictly to the ethical standards outlined in the Declaration of Helsinki (47), along with the guidelines for Good Clinical Practice recommended by the International Council for Harmonization (48), thereby ensuring the safety and rights of all participants are safeguarded. Ethical approval has been obtained from the research ethics committee of the Hospital of Chengdu University of Traditional Chinese Medicine (approval number: 2024KL-112). Obtaining written informed consent from all participants is a prerequisite for their involvement in any part of the study. After the study concludes, the findings will be shared through publication in a scholarly peer-reviewed journal.
